# The complete chloroplast genome of *Acanthus ilicifolius*, an excellent mangrove plant

**DOI:** 10.1080/23802359.2021.1884022

**Published:** 2021-04-04

**Authors:** Li Xu, Xin-Rui Wang, Kuo Sun, Ting Yu, Jiu-Heng Xu, Ping-Xing Ding, Li-Ming Tang, Dong-Xu Zhang, Wen-Bin Guan

**Affiliations:** a School of Ecology and Nature Conservation, Beijing Forestry University, Beijing, China; b Forestry Department of Guangxi, Nanning, China; c Protected Agricultural Technology Development Center, Shanxi Datong University, Datong, China

**Keywords:** *Acanthus ilicifolius*, Acanthaceae, chloroplast genome, phylogenetic analysis

## Abstract

*Acanthus ilicifolius* is an excellent mangrove plant. In this study, the complete chloroplast genome of *A. ilicifolius*, a salt tolerant plant of Acanthaceae, was generated. The length of chloroplast genome is 150,758 bp, in which the large-single copy region (LSC) is 82,963 bp, the small-single copy (SSC) region is 17,191 bp, and a pair of inverted repeat (IRa and IRb) regions is 25,302 bp. The chloroplast genome contains 128 genes, including 84 protein-coding genes, eight rRNA genes, and 36 tRNAs genes, with a total GC content of 38%. Phylogenetic analysis showed that *A. ilicifolius* was closely related to *A. ebracteatus*, both species belonged to *Acanthus* genus.

*Acanthus ilicifolius* is a shrubby plant of Acanthaceae, which mainly distributed in tropical coastal areas and is one of the important components of mangroves. It is a medicinal plant with antioxidant, analgesic, and anti-inflammatory effects that can be used to treat various diseases of people (Chi et al. 2019). To date, the research on *A. ilicifolius* has mainly focused on its geographical distribution, population characteristics, medicinal value, physiological and biochemical aspects, and there is no report on the chloroplast genome of *A. ilicifolius.* Here, we assembled the complete chloroplast genome of *A. ilicifolius* and performed phylogenetic analysis with the chloroplast genomes of other species of Acanthaceae, in order to explore its phylogenetic relationship and provide a basis for molecular biology research.

The leaves of *A. ilicifolius* were collected from Xiangzhou District, Zhuhai City, Guangdong Province, China (N 22°25′44.99″, E 113°38′25.75″). The voucher specimen was deposited in the Herbarium of Beijing Forestry University (BJFC) (under collection numbers of GWBAI001). Chloroplast sequences were obtained by high-throughput sequencing and splicing of genomic data (Yu et al. 2011). The genomic DNA was extracted using Plant Genomic DNA Kit (DP305), which was manufactured to average 150 bp paired-end (PE) library and sequenced on the Illumina Novaseq Sequencing platform. Filtered chloroplast reads were exploited for *de novo* assembly with SPAdes v3.10.1 (Antipov et al. 2016). The chloroplast sequences were annotated by CpGAVAS (Liu et al. 2012), and the annotations were verified by Geneious Prime (Kearse et al. 2012).

The chloroplast genome of *A. ilicifolius* (GenBank accession no. MW174172) was a circular molecular genome with a size of 150,758 bp in length and had four distinct parts. The large-single copy (LSC) region with 82,963 bp and small-single copy (SSC) region with 17,191 bp are separated by a pair of inverted repeat regions with 25,302 bp. It contained 128 genes (84 protein-coding genes, eight rRNA genes, and 36 tRNA genes). The overall GC content of the chloroplast genome is 38.0% and those in the LSC, SSC, and IR regions are 36.4%, 32.3%, and 43.7%, respectively.

In order to understand the phylogenetic relationship between *A. ilicifolius* and other species of Acanthaceae, the complete chloroplast genome sequences of 16 species were downloaded from NCBI GenBank database. Sequences were aligned by MAFFT (Katoh et al. 2002), and a phylogenetic tree was constructed using the maximum-likelihood (ML) method with 1000 bootstrap values in the PhyloSuite (Zhang et al. 2020). At the same time, we used the ML method in MEGA7.0 (Kumar et al. 2016) to verify the phylogenetic relationship and results show consistency. Phylogenetic analysis showed that the Acanthaceae could be divided into two clades. One of the clades contains *Andrographis*, *Barleria*, *Echinacanthus*, *Strobilanthes*, *Clinacanthus*, *Rungia*, and *Justicia*. The other clade contains *Aphelandra*, *Acanthus*, and *Blepharis*. *A. ilicifolius* is sister to *A. ebracteatus*. *Acanthus* and *Blepharis* are grouped together and closely related to *Aphelandra* (Figure 1). The complete chloroplast genome of *A. ilicifolius* can provide a reference for further study on the phylogeny and evolution of Acanthaceae plants, as well as for the protection and utilization of these multifunctional natural resources.

**Figure 1.**
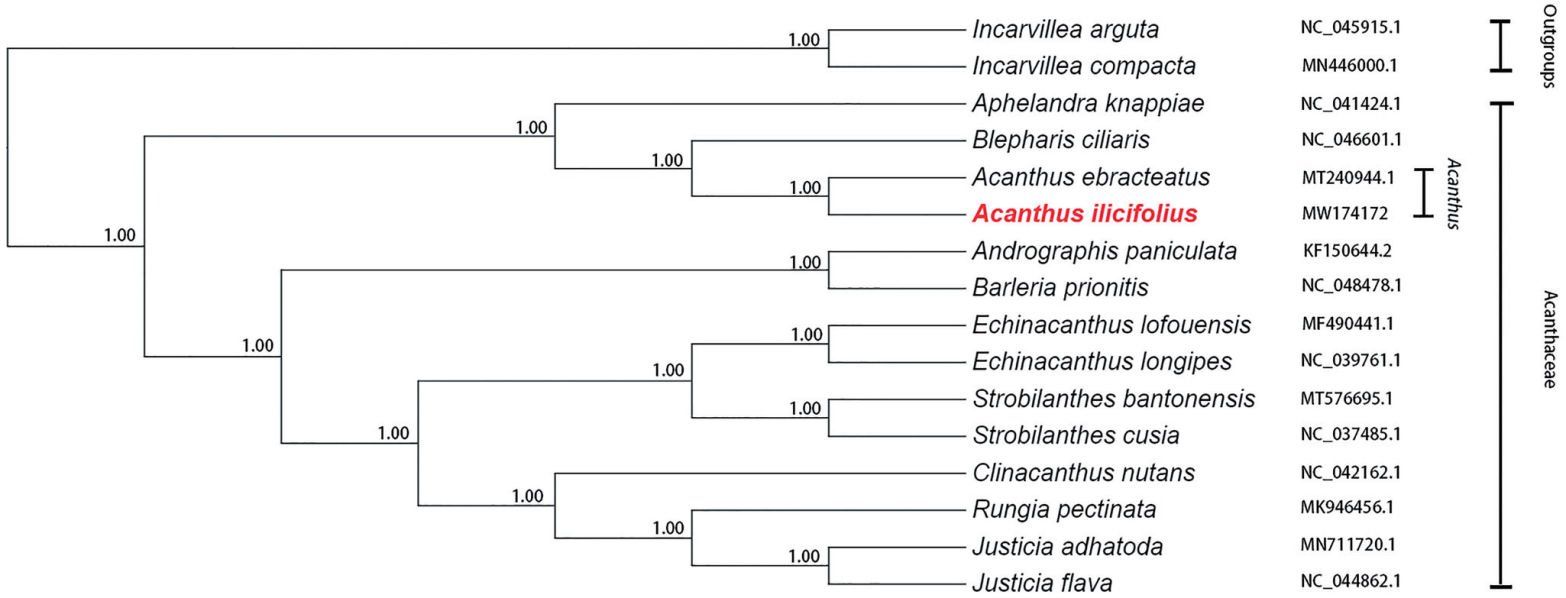
Maximum-likelihood phylogenetic tree inferred from 16 complete chloroplast genome sequences. The position of *A. ilicifolius* is marked in red and bootstrap values are listed for each branch.

## Data Availability

The data that support the findings of this study are openly available in NCBI at https://www.ncbi.nlm.nih.gov/nuccore/MW174172, reference number MW174172.
